# Vitamin A-Mediated Birth Defects: A Narrative Review

**DOI:** 10.7759/cureus.50513

**Published:** 2023-12-14

**Authors:** Raegan B Abadie, Abigail A Staples, Lillian V Lauck, Alexandra D Dautel, Noah J Spillers, Rachel J Klapper, Jon D Hirsch, Giustino Varrassi, Shahab Ahmadzadeh, Sahar Shekoohi, Alan D Kaye

**Affiliations:** 1 School of Medicine, Louisiana State University Health Sciences Center, Shreveport, USA; 2 Radiology, Louisiana State University Health Sciences Center, Shreveport, USA; 3 Pain Medicine, Paolo Procacci Foundation, Rome, ITA; 4 Anesthesiology, Louisiana State University Health Sciences Center, Shreveport, USA

**Keywords:** current recommendations, pregnant intake, birth defects, excess, provitamin, teratogenicity, vitamin a

## Abstract

Vitamin A deficiency (VAD) or excess in expectant mothers can result in fetal abnormalities such as night blindness, bone anomalies, or epithelial cell problems. In contrast, excessive vitamin A in pregnancy can precipitate fetal central nervous system deformities. During pregnancy, a pregnant woman should monitor her vitamin A intake ensuring she gets the recommended dosage, but also ensuring she doesn't exceed the recommended dosage, because either one can result in teratogenicity in the fetus. The widespread and unregulated use of multivitamins and supplements makes consuming doses greater than the recommended quantity more common in developed countries. While vitamin A excess is more common in developed countries, deficiency is most prevalent in developing countries. With proper maintenance, regulation, and education about VAD and excess, a pregnant mother can diminish potential harm to her fetus and potential teratogenic risks.

## Introduction and background

Vitamin A is a fat-soluble vitamin essential in the normal nonpathogenic functioning of many human body processes. It plays an important role in eye development, prevention of blindness, bone development, skin and mucosa protection, and immunity, and aids in the development of epithelial tissue, such as teeth and hair. In addition to these important functions, it is also essential for standard embryo development [1]. During pregnancy, a mother’s nutrient requirement increases to maintain proper fetal development. This requirement is especially important during the third trimester when the fetus undergoes rapid growth and needs the developmental benefits of vitamin A [2-4]. Many developing countries have mothers who experience vitamin A deficiency (VAD), leading to abnormal development of the fetus. This deficiency can be due to socioeconomic factors since most deficiencies are of higher prevalence in poor, underserved, countries with inequalities in income, education, housing, and access to medical care [2]. If an expectant mother has an excess intake of vitamin A during the first trimester, central nervous system derangements, cardiovascular system abnormalities, or spontaneous abortions may result [2-4]. This is because high serum levels of retinoic acid, one of the three forms of vitamin A, interfere with genes essential for the development of the fetus. A pregnant woman should watch the levels of vitamin A that she consumes because any extra can result in teratogenicity in the fetus, this can occur through education about what foods or supplements contain vitamin A, and monitoring their intake of such foods. The World Health Organization (WHO) recommends a maximum dose of 10,000 IU daily during the first 60 days of fetal development when crucial and susceptible development occurs for all mothers [2-4]. Because of the dangers of both vitamin A excess and deficiency, it is important that levels are monitored during pregnancy to ensure the mother is maintaining an appropriate level for her fetus.

## Review

Vitamin A overview

Vitamin A is a fat-soluble vitamin found naturally in many foods, including fruits, vegetables, and animal-based products. It is crucial for the normal functioning of many distinct functions of the human body, such as visual adaptation, immunity, and epithelial cell differentiation [5]. Once ingested, it must be metabolized into its biologically active forms, which include retinol, retinal, and retinoic acid, with the most prominent forms being 11-cis-retinal and all-trans-retinoic acid (ATRA) [5]. Each form has different roles in the human body [5]. While it may be administered topically or as an injectable, vitamin A is predominantly taken orally and absorbed by enterocytes after metabolizing it into retinol. Once absorbed, it can be transported to other cells bound to cellular retinol-binding protein. Its main target is the liver, where it is stored as retinyl palmitate [5,6], and its absorption is increased with increased fat intake through the diet [5]. The kidneys eliminate vitamin A derivatives through urine or the liver through bile [5]. Because it is fat-soluble and stored in the liver, it can take months before deficiency becomes evident. On the other hand, toxicity can occur easily with excess intake or altered metabolism. Intake varies depending on sex and status of pregnancy and/or lactation and is measured in retinol activity equivalent (RAE), where one RAE equals retinol 1 microgram [6]. The recommended daily allowance is 900 micrograms per day for males, 700 micrograms per day for non-pregnant and non-lactating females, 750-770 micrograms per day for pregnant females over the age of 18, and 1300 micrograms per day for lactating females over the age of 18; regardless of sex or pregnancy and lactation status, intake should not exceed 3000 micrograms per day [6]. The serum concentration of vitamin A in adults ranges from 300 to 700 ng/mL with a peak plasma time of four to five hours in oil solution or 304 hours when water-miscible [6]. Vitamin A and its levels can be impacted by estrogens and oral contraceptives, increasing retinol-binding protein levels [5]. In addition, alcohol damages the liver over time and decreases the amount of stored vitamin A [5].

Current uses of vitamin A 

Vitamin A is found naturally in many food items, including liver, butter, other dairy products, eggs, chicken, beef, fish, and certain vegetables such as carrots and sweet potatoes. In addition to its natural sources, vitamin A and its derivatives can also be found in over-the-counter supplements and are prescribed as a treatment for certain medical conditions [7]. If a pregnant mother was educated on which foods contain vitamin A, she could easily monitor her intake. Vitamin A supplementation is crucial in individuals who do not receive adequate intake through diet alone, which is uncommon in developed countries. Sufficient intake is necessary to prevent symptoms of VAD, such as night blindness, dry skin, keratomalacia, and immunosuppression. In addition to its benefits for development and metabolic processes, vitamin A has important pharmaceutical benefits. One commonly prescribed medication is isotretinoin, a vitamin A-derived medication administered orally over months to treat severe cystic acne [8]. Because vitamin A also plays a role in adaptive immunity, its supplementation in children is part of the supportive treatment plan for measles [9]. Similarly, supplementation of vitamin A may be an option for treating severe acute respiratory syndrome coronavirus 2 [10]. Treatment modalities in these cases include a topical nasal application for anosmia and oral supplements in combination with steroids for patients with severe active infection. Individuals recovering from the virus may also benefit from oral vitamin A supplementation [10].

Teratogen effects

Studies have examined the relationship between vitamin A use in pregnancy, specific organ outcomes, and developmental anomalies. One meta-analysis of six studies examined the association between vitamin A during pregnancy and nonsyndromic orofacial clefts. More specifically, the study explored vitamin A use during the periconceptional period, or three months before conception, and the first trimester. Results demonstrated a significant protective effect of periconceptional vitamin A on the nonsyndromic cleft lip with or without cleft palate (NSCL/P) but a nonsignificant protective effect for nonsyndromic cleft palate only. It has been posited that excess or deficient levels of vitamin A during pregnancy can affect the development and growth of the kidneys and urinary tract in offspring. Previous studies exhibited smaller kidneys in the offspring of women with deficient levels of vitamin A during pregnancy [11,12]. Ozisik et al. found a significant shared commonality in vitamin A target genes and genes known to cause congenital anomalies of the kidney and urinary tract [11,12]. While this finding suggests that vitamin A plays a role in cell signaling, growth, and development of the kidneys and urinary tract, further research is necessary to study specifics, including periods of development that may be more vulnerable to variations in vitamin A and doses [11,12].

VAD is most common in developing countries. In contrast, excess vitamin A consumption, possibly teratogenic, is more prevalent in developed countries [2]. The widespread and unregulated use of multivitamins and supplements in developed countries, for example, vitamin A supplements, makes consuming doses greater than the recommended quantity much easier. This is partially due to supplements not being held to the same regulations as pharmaceuticals in the United States, and patients should always consult a physician before beginning any supplement regimen, especially during pregnancy [4]. The WHO defines VAD as <0.70 µmol/L serum retinol levels [2]. During the third trimester, VAD becomes a concern when maternal blood volume and fetal development rate increase [2]. One study evaluated the effects of chemically induced maternal VAD in mice and its effect on the development of congenital diaphragmatic hernias (CDH). Abnormalities in the retinoid signaling pathway are one mechanism proposed to contribute to CDH. Results showed that low levels of maternal vitamin A increased the incidence of teratogen-induced CDH [13]. VAD is known to have dangerous effects on the immune system, ocular health, and growth and development of children. VAD, low serum retinol, and low insulin-like growth factor 1 (IGF-1) are thought to contribute to a poor immune system and poor growth and development in children with Down syndrome (DS). A cross-sectional study on 47 children ages 24-72 months with DS revealed a prevalence of VAD of 25.5% (n = 12; 95%CI: 13.9-40.3) [14]. The relative dose-response test determined VAD results that suggested low vitamin A stores in the liver. About 74.5% of children had serum retinol levels <0.70 µmol/L and there was no significant association between VAD and IGF-1 levels, there was a positive correlation between retinol levels and IGF-1 levels [14].

While vitamin A was shown to be protective against NSCL/P [11], another study using mouse embryonic palatal mesenchymal cells investigated the effects of the vitamin A derivative ATRA on their differentiation and mineralization. The results illustrated that ATRA inhibited Wnt signaling, therefore impeding bone formation. This demonstrates a possible connection between ATRA and cleft lips/palates, though further animal and human models are necessary to continue exploring this relationship [2]. Vitamin A plays a role in the development of pharyngeal arch arteries, cranial nerves, and hindbrain, which all function in swallowing. Excess maternal vitamin A in mouse models of 22q11.2 deletion syndrome resulted in pups with exacerbated abnormalities of the fourth pharyngeal arch artery, cranial nerve V, and expression of Cyp26b1 in the hindbrain that contributed to swallowing difficulties as well as increased lung inflammation, a sign of aspiration [15,16]. Doses over 10,000 IU a day are thought to have the potential for teratogenic risks, especially if excessive intake occurs during the first quarter of the pregnancy [2]. Studies have shown that doses exceeding this amount affect the development of neural crest tissues, urinary tract, and cardiac structures [2].

Vitamin A's current recommendations

Although it is still unclear whether specific amounts of these metabolites are generated with each vitamin A supplement, 13-cis-retinoic acid is the main teratogen of Isotretinoin, a common treatment for cystic acne [2]. Because of the body's variability in metabolism and lack of clinical examples, there is a paucity of data on doses of vitamin A that establish a threshold of teratogenicity. It is generally assumed that when doses rise above 10,000 IU per day of vitamin A in a pregnant woman with baseline normal vitamin A levels, there is a potential risk of teratogenicity, and reports suggest that fetuses of pregnant mothers taking doses greater than 25,000 IU/day had urinary tract malformations [17,18]. According to the WHO, supplementation is not recommended in developed countries with a nutritionally adequate diet, and pregnant mothers should consume a dietary allowance of 2670 IU of vitamin A. Currently, guidelines indicate a maximum of up to 10,000 IU daily before 60 days of gestation and no more than 25,000 IU weekly after 60 days. This difference is due to the higher risk of early pregnancy effects which can be seen in Table 1 [19-24]. Suppose a pregnant woman's intake of retinol exceeds 10,000 IU per day. In that case, the American Heart Association recommends that fetal echocardiography be obtained during the prenatal period due to the risk for cardiomyopathy with a minimal absolute risk between 1% and 2% [3].

**Table 1:** Clinical efficacy

Author	Groups studied and intervention	Results and findings	Conclusions
Study 1: Amaeze et al. [19]	A randomized placebo-controlled trial assessed the changes in CYP2D6 and CYP3A activities throughout pregnancy and studied the effect of vitamin A on CYP2D6.	The Dextrorphan (DX)/ 3-hydroxymophinan (3HM)/ Dextromethorphan (DM) urine ratios used to assess increased CYP3A and CYP2D6 activity were significantly higher during pregnancy compared to post-partum. Vitamin A supplements did not change CYP2D6 activity however plasma all-trans retinoic acid concentrations positively correlated with increased CYP2D6 activity during pregnancy and postpartum.	Further research should be done to look at the mechanism of increased CYP2D6 activity during pregnancy.
Study 2: Ding et al. [20]	A randomized placebo-controlled trial evaluated the effects of daily oral low-dose vitamin A supplementation in lactating mothers and the health status of infants in China.	While maternal serum retinol concentration is usually expected to drop during breastfeeding, the decrease in the supplemented group had a significantly lower drop than in the control group. Additionally, maternal serum retinol concentrations were shown to increase in the supplementation group, with no change observed in the control group. Infant febrile illness, respiratory tract infections, diarrhea, and eczema showed no difference between the two groups despite vitamin A supplementation.	Daily oral vitamin A supplementation helps improve vitamin A status. However, there was no effect on infant health status through breast milk.
Study 3: Nga et al. [22]	A randomized controlled trial assessed the effects of a nutrient-rich, food-based supplement given to rural Vietnamese mothers before and/or during pregnancy on birth outcomes.	The food-based supplement given from pre-conception to term or mid-gestation to term increased protein, iron, zinc, folate, vitamin A, and B12 intakes; however, it failed to alter infant measurements such as height and weight, at birth.	A nutrient-rich, food-based supplement did not affect maternal or infant outcomes.
Study 4: Hidayat et al. [23]	A randomized clinical trial to evaluate b-hCG levels in low-risk gestational trophoblastic neoplasia (GTN) patients after vitamin A administration.	Compared to control groups, levels of b-hCG and incidence of chemotherapy resistance were found to be lower for patients with low-risk GTN who underwent methotrexate chemotherapy.	For low-risk GTN patients undergoing methotrexate chemotherapy, an oral dose of 6,000 IU of vitamin A given orally could help to reduce b-hCG levels.
Study 5: Palmer et al. [24]	A cluster-randomized study focusing on how nutritional content i.e., vitamin A or β-carotene affects thymus phenotype during prenatal and childhood in rural Nepal.	There is a positive correlation between gestational age at delivery and higher thymulin concentrations among children born to β-carotene-supplemented mothers. Height and weight of children between the ages of 9-12 years showed a positive correlation to thymulin concentrations as well. Seasonal decreases in thymulin levels were observed during pre-monsoon, monsoon, and pre-harvest relative to the post-harvest season.	Findings give a baseline on how nutritional content affects the phenotyping of the thymus in prenatal and adolescence. The potential disease risk of undernutrition could also affect the role of thymulin in the neuroendocrine regulation of inflammation, but this has yet to be explored.
Study 6: Haskell et al. [21]	Two randomized trials assess small-quantity, lipid-based nutrient effects on plasma or milk retinol concentration among young Malawian or Ghanaian children.	Plasma retinol concentrations and milk retinol concentrations in Malawian mothers did not change between the groups that received iron and folic acid supplements, multiple micronutrients, and small-quantity lipid-based nutrient supplements. A similar result was found in Malawian children and Ghanaian children.	Small-quantity lipid-based nutrient supplements have no effect on vitamin A levels in Malawian mothers and Malawian and Ghanaian children.

Clinical trials related to vitamin A toxicity

More research is required to better understand vitamin A’s mechanisms and effects, especially during the prenatal period. For example, factors causing increased CYP2D6, an enzyme important for drug metabolism, and its activity during pregnancy, as well as the role of undernutrition on thymulin in neuroendocrine regulation of inflammation, require additional study as seen in Table 1 [19,24]. Other studies attempting to ascertain the effects of vitamin A yielded contrasting results. Notably, in Ding et al., daily oral vitamin A supplementation helped improve vitamin A status in many pregnant women who are deficient [20], while in Haskell et al., small-quantity lipid-based nutrient supplements did not affect vitamin A levels [21]. Regardless, in these two studies, along with Nga et al., various versions of vitamin A supplementation did not affect maternal or infant outcomes as seen in Table 1 [22]. While this research provides some insight into the role of vitamin A in the prenatal period, it also demonstrates the necessity of further exploration into these relationships.

Discussion

According to current evidence, it is essential that pregnant mothers maintain recommended levels of vitamin A as recommended by WHO throughout pregnancy to ensure their health and the health of their baby. Vitamin A is a fat-soluble vitamin found in many foods, fruits, vegetables, and animal-based products. It is important for visual acuity, immunity, bone growth, and epithelial cell differentiation and therefore very important in the development of fetuses [5,25,26]. In developed countries, vitamin A supplementation is critical in patients who do not receive satisfactory consumption through their diet. Appropriate levels of vitamin A are necessary to prevent symptoms of VAD, such as night blindness, dry skin, keratomalacia, and immunosuppression. One of the most common uses of prescribed vitamin A isotretinoin is a vitamin A-derived medication administered orally over months for treating severe cystic acne; however, it has a teratogenic association [8,27]. Many individuals may start medication for their acne without realizing the teratogenic effects that it will have on their future child. Results from trials assessing the teratogenicity of vitamin A have demonstrated that it affects gene signaling and inhibits bone formation, resulting in cleft lips or palate [11,28]. It has also been shown that excess can lead to abnormalities in pharyngeal arches, which results in impaired swallowing and aspiration [16]. However, deformities in the fetus can also occur in deficient levels of vitamin A, which is why individuals often take vitamin A supplementation. Vitamin A levels should be monitored very carefully to prevent teratogenic effects. Pregnancy increases the body’s nutritional requirements, including micronutrients such as vitamin A. There is a higher incidence of excessive vitamin A consumption in developed countries, whereas, in developing countries, VAD is more common [2,29]. However, both the excessive and deficient levels of vitamin A result in a negative effect on the fetus. With proper education to patients, vitamin A teratogenicity can be avoided and therefore result in a healthier baby.

## Conclusions

The pregnant mother must weigh the need for an adequate supply of vitamin A to avoid deficiency against excess consumption, potentially leading to teratogenicity. The unregulated use and lack of education on the teratogenic effects of vitamin A multivitamins and supplements in developed countries make it much easier to consume doses greater than the recommended quantity by the WHO. Mothers who consumed more than 25,000 IU/day of vitamin A had children with an increased risk of urinary tract malformations. However, the recommendation is no more than 10,000 IU daily before 60 days of gestation and no more than 25,000 IU weekly to prevent the teratogenicity risk. Educating expectant mothers on the risks associated with both deficient and excessive levels of vitamin A can prevent deformities related to vitamin A malnutrition in the child.
